# A complex of the ubiquitin ligase TRIM32 and the deubiquitinase USP7 balances the level of c-Myc ubiquitination and thereby determines neural stem cell fate specification

**DOI:** 10.1038/s41418-018-0144-1

**Published:** 2018-06-13

**Authors:** Sarah Nicklas, Anna-Lena Hillje, Satoshi Okawa, Ina-Maria Rudolph, Franziska Melanie Collmann, Thea van Wuellen, Antonio del Sol, Jens C. Schwamborn

**Affiliations:** 1 Developmental and Cellular Biology Group, Luxembourg Centre for Systems Biomedicine (LCSB), University of Luxembourg, L-4367 Belvaux, Luxembourg; 2 Stem Cell Biology and Regeneration Group, Institute of Cell Biology, ZMBE, Westfälische Wilhelms-Universität Münster, 48149 Münster, Germany; 3 Computational Biology Group, Luxembourg Centre for Systems Biomedicine (LCSB), University of Luxembourg, L-4367 Belvaux, Luxembourg

**Keywords:** Cell biology, Neuroscience

## Abstract

The balance between stem cell maintenance and differentiation has been proposed to depend on antagonizing ubiquitination and deubiquitination reactions of key stem cell transcription factors (SCTFs) mediated by pairs of E3 ubiquitin ligases and deubiquitinating enzymes. Accordingly, increased ubiquitination results in proteasomal degradation of the SCTF, thereby inducing cellular differentiation, whereas increased deubiquitination stabilizes the SCTF, leading to maintenance of the stem cell fate. In neural stem cells, one of the key SCTFs is c-Myc. Previously, it has been shown that c-Myc is ubiquitinated by the E3 ligase TRIM32, thereby targeting c-Myc for proteasomal degradation and inducing neuronal differentiation. Accordingly, TRIM32 becomes upregulated during adult neurogenesis. This upregulation is accompanied by subcellular translocation of TRIM32 from the cytoplasm of neuroblasts to the nucleus of neurons. However, we observed that a subpopulation of proliferative type C cells already contains nuclear TRIM32. As these cells do not undergo neuronal differentiation, despite containing TRIM32 in the nucleus, where it can ubiquitinate c-Myc, we hypothesize that antagonizing factors, specifically deubiquitinating enzymes, are present in these particular cells. Here we show that TRIM32 associates with the deubiquitination enzyme USP7, which previously has been implicated in neural stem cell maintenance. USP7 and TRIM32 were found to exhibit a dynamic and partially overlapping expression pattern during neuronal differentiation both in vitro and in vivo. Most importantly, we are able to demonstrate that USP7 deubiquitinates and thereby stabilizes c-Myc and that this function is required to maintain neural stem cell fate. Accordingly, we propose the balanced ubiquitination and deubiquitination of c-Myc by TRIM32 and USP7 as a novel mechanism for stem cell fate determination.

## Introduction

Stem cells are defined by the following two characteristics: they are able to self-renew, thereby maintaining the stem cell pool, or to undergo differentiation, thereby generating more specialized cell types [1]. The balance between stem cell maintenance and differentiation requires tight control at various levels, including post-translational regulation of key proteins. One of the major forms of post-translational protein modifications in eukaryotes is ubiquitination, which serves as a key regulatory mechanism to control the stability and activity of proteins [2–5].

It has been previously proposed, that the fate specification of stem cells is controlled by antagonizing ubiquitination and deubiquitination of so-called stem cell transcription factors (SCTFs) [6, 7]. According to this hypothesis, each SCTF is post-translationally regulated by at least one pair of specific E3 ligase and DUB. Dominant E3 ligase activity will result in ubiquitination of the SCTF, thereby targeting it for proteasomal degradation and releasing its repression of differentiation-inducing genes, ultimately leading to cell differentiation. In contrast, dominant DUB activity will lead to deubiquitination and stabilization of the SCTF, which in turn suppresses the expression of differentiation-inducing genes, thus resulting in stem cell maintenance [6, 7]. The net balance of ubiquitination and deubiquitination of SCTFs hence determines the fate the stem cell will adopt.

Neural stem cells (NSCs) in the adult mammalian brain reside in two neurogenic niches, the subventricular zone (SVZ) lining the walls of the lateral ventricle, as well as the subgranular zone (SGZ) in the dentate gyrus of the hippocampus [1]. In the SVZ, adult NSCs (type B cells) proliferate and give rise to rapidly dividing transient amplifying cells (type C cells). These type C cells differentiate into neuroblasts (type A cells) that migrate in chains along the rostral migratory stream (RMS) towards the olfactory bulb (OB). Once they reach the OB, neuroblasts maturate into fully functional neurons, which become integrated into the existing neuronal networks [8, 9]. This process of generating functional neurons from NSCs is called neurogenesis.

One of the key SCTFs in NSCs is c-Myc. c-Myc is a master transcriptional regulator in many cell types, controlling the expression of numerous genes implicated in various cellular processes, most importantly in cell proliferation, growth and stem cell renewal. Aberrant overexpression of c-Myc is often associated with the development of malignancies and tumorigenesis [10, 11]. In contrast, depletion of c-Myc in mice leads to early embryonic lethality between E9.5 and E10.5 [12], demonstrating the necessity of tightly controlled c-Myc levels. We previously have shown, that in NSCs c-Myc levels are controlled by the E3 ligase TRIM32, which ubiquitinates c-Myc and thereby targets it for proteasomal degradation [13, 14]. High levels of c-Myc have been shown to be crucial for NSC self-renewal and for maintaining an undifferentiated phenotype [15, 16]. Hence, the TRIM32-mediated reduction in c-Myc levels is essential for inducing cell cycle exit and neuronal differentiation in NSCs of the embryonic [13, 14, 17] and adult mouse brain [18]. TRIM32 is a cell fate determinant that becomes upregulated during adult neurogenesis from being absent or weakly expressed in type B and C cells of the SVZ to being highly expressed in mature OB neurons. Along with its upregulation, TRIM32 translocates from the cytoplasm of neuroblasts within the RMS to the nucleus of neurons within the OB [18]. It is tempting to speculate, that this nuclear translocation of TRIM32 is required for enabling the ubiquitination of c-Myc and thus for inducing neuronal differentiation.

By making use of a recently performed mass spectrometry screen [19], we here identify the DUB USP7 as novel TRIM32 interaction partner. In addition, we provide a detailed expression analysis of USP7 in the neural system in vitro and in vivo and found that TRIM32 and USP7 co-localize in NSCs and neurons in vitro, but show a rather dynamic expression pattern in the mouse SVZ-RMS-OB system in vivo. We are further able to demonstrate that USP7 stabilizes and deubiquitinates c-Myc, that has been poly-ubiquitinated by TRIM32, demonstrating that USP7 indeed antagonizes TRIM32 activity. Most importantly, inhibiting USP7 activity not only led to a destabilization of c-Myc in NSCs and neurons, but also to decreased expression of the NSC marker Sox2. This strongly indicates that the USP7 function is indeed required for the maintenance of NSC fate and for preventing neuronal differentiation. Thus, balanced ubiquitination and deubiquitination of c-Myc by TRIM32 and USP7 may represent a novel mechanism by which NSCs determine cell fate decisions during adult neurogenesis.

## Results

### TRIM32 is expressed in the nucleus of a subpopulation of proliferating transient amplifying cells

Previously, we have shown that the cell-fate determinant TRIM32 becomes upregulated during neuronal differentiation of mouse NSCs in vitro [13, 19] as well as during mouse embryonic [14] and adult neurogenesis in vivo [18]. This upregulation is accompanied by subcellular translocation of TRIM32 from the cytoplasm of progenitor cells to the nucleus of mature neurons [13, 14, 18, 19] (Supplementary Fig. 1a), where it presumably functions to target c-Myc for proteasomal degradation (Supplementary Fig. 1b and c) [13, 14].

**Fig. 1:**
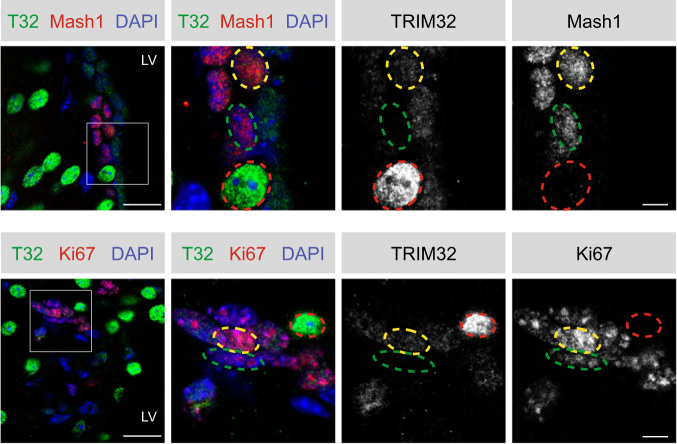
Subcellular localization of TRIM32 in proliferating cells of the SVZ. Immunostainings of adult mouse brain sections with the indicated antibodies. Images were taken in the SVZ region. The right panels show a higher magnification of the boxed areas. Cells expressing high levels of TRIM32 are highlighted in red, cells expressing low levels of TRIM32 are highlighted in yellow and cells lacking TRIM32 expression are highlighted in green. Scale bars = 20 µm; for high magnifications, 5 µm; *N* = 3. LV lateral ventricle

Consistently, a subset of cells in the adult mouse SVZ were found to express high levels of TRIM32 in the nucleus, while being negative for the cell cycle marker Ki67 and the transient amplifying (type C) cell marker Mash1 (Fig. 1, red circles) and hence most likely represent mature neurons. Interestingly, we further observed a subpopulation of cells expressing low levels of TRIM32 in the nucleus (Fig. 1, yellow circles), which also co-expressed Ki67 and Mash1 (Fig. 1). However, not all of the Ki67 + or Mash1 + cells were found to co-express TRIM32 in the nucleus (Fig. 1, green circles), indicating that TRIM32 is present in the nucleus of only a subset of proliferating type C cells. Since type C cells are highly proliferative and do not yet undergo neuronal differentiation, we hypothesized that the ubiquitination function of TRIM32 towards c-Myc, which ultimately induces neuronal differentiation, has to be antagonized in these cells.

### Identification of USP7 as a novel TRIM32-interacting protein

**Fig. 2:**
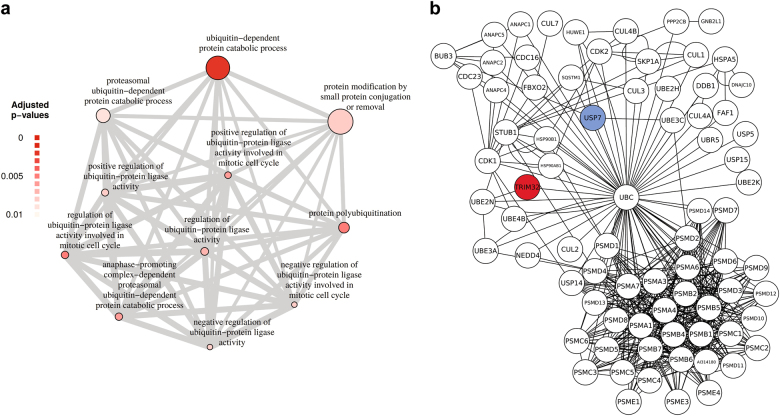
Identification of ubiquitination-related proteins associated with TRIM32 via mass spectrometry. **a** Enriched biological process GO term analysis (adjusted *p* ≤ 0.01) of TRIM32-associated proteins that were significantly more abundant compared to the negative control with a focus on processes associated with ubiquitination. Each circle represents an enriched GO term and the thickness of lines between circles correlates with the number of common genes between two GO categories. Circle size correlates with the number of genes and the circle color gradient indicates the GO enrichment significance level based on the adjusted *p* value. **b** Network analysis of TRIM32-associated proteins affiliated with the enriched GO term “ubiquitin-dependent protein catabolic process”, shown in (**a**). The red circle depicts the TRIM32 bait protein, white circles are significantly enriched TRIM32-associated proteins over the negative control, of which USP7 (highlighted in blue) was chosen for further analysis. Protein–protein interaction knowledge was retrieved from MetaCore

In order to identify proteins that may antagonize the ubiquitination function of TRIM32, we made use of a list of potential TRIM32 interaction partners, which we had previously identified in a mass spectrometry screen [19]. We performed a GO enrichment analysis focussing on biological processes that are associated with ubiquitination (Fig. 2a). Network analysis of the GO term with the highest significance level, “ubiquitin-dependent protein catabolic processes”, revealed several deubiquitinating enzymes as potential TRIM32 interaction partners (Fig. 2b). Out of these, the DUB USP7 is of special interest, as it has been implicated in neural progenitor cell maintenance before [6, 7].

**Fig. 3:**
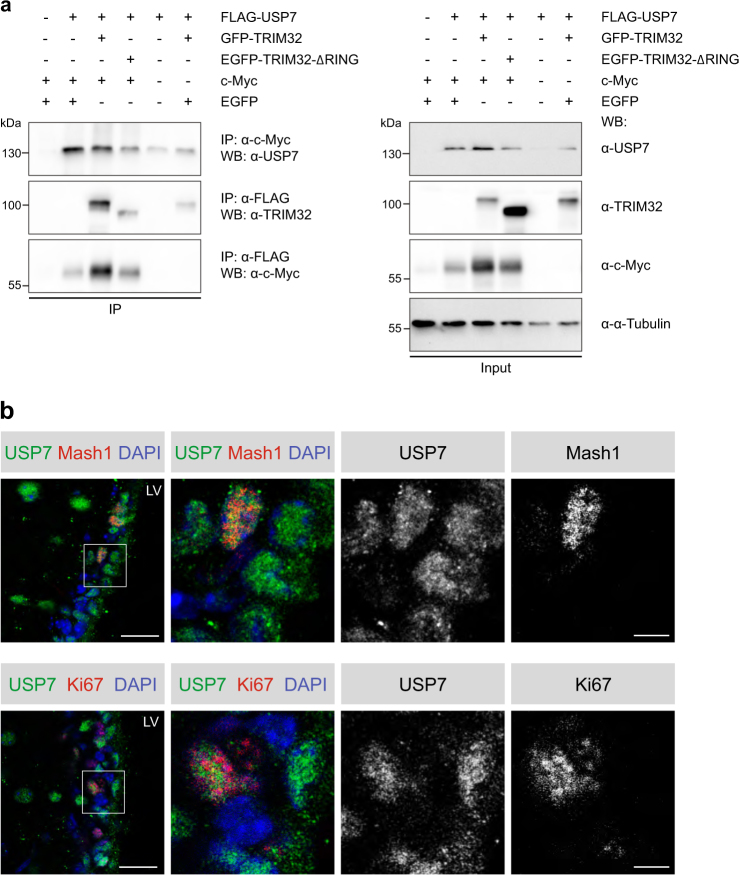
TRIM32, USP7 and c-Myc directly interact with each other and USP7 shows the same expression pattern as TRIM32 in proliferating cells of the SVZ. **a** HEK293T cells were transfected with the indicated constructs. FLAG-tagged USP7 was immunoprecipitated with an anti-FLAG antibody, while c-Myc was immunoprecipitated with an anti-c-Myc antibody. USP7, TRIM32 and c-Myc were detected with specific antibodies as indicated for the immunoprecipitation (IP) and the lysate fraction. An α-Tubulin Western blot was used as loading control. *N* = 3. **b** Immunostainings of adult mouse brain sections with the indicated antibodies. Images were taken in the SVZ region. The right panels show a higher magnification of the boxed areas. Scale bars = 20 µm; for high magnifications, 5 µm; *N* = 3. LV lateral ventricle, EGFP enhanced green fluorescent protein, IP immunoprecipitation, WB western blot

To confirm the interaction between TRIM32 and USP7, FLAG-tagged USP7 was co-expressed with GFP-tagged TRIM32 in HEK293T cells and immunoprecipitated using an anti-FLAG antibody, leading to co-precipitation of GFP-TRIM32 (Fig. 3a, IP lane 3). A deletion construct of TRIM32 lacking the RING domain (pEGFP-TRIM32-ΔRING) was still able to co-immunoprecipitate FLAG-USP7, albeit less efficiently (Fig. 3a, IP lane 4), demonstrating that the RING domain of TRIM32 is not required for the interaction with USP7, but might enhance it. Furthermore, FLAG-USP7 was able to co-immunoprecipitate overexpressed c-Myc (Fig. 3a), while c-Myc vice versa also co-precipitated FLAG-USP7 (Fig. 3a). Noteworthy, c-Myc is barely detectable when overexpressed in the absence of USP7 (Fig. 3a, Input lane 1 versus lanes 2–4), suggesting that USP7 is able to stabilize c-Myc.

To gain evidence that these interactions are relevant for the regulation of NSC fate in vivo, we analyzed the expression pattern of USP7 in proliferating type C cells in the SVZ of adult mice. Interestingly, we found USP7 to be expressed in some, but not all, Mash1- and Ki67-expressing cells in the SVZ (Fig. 3b), similar to the expression pattern observed for TRIM32 (Fig. 1). It is tempting to speculate, that the subpopulation of type C cells expressing low levels of TRIM32 in the nucleus overlaps with the subpopulation of type C cells expressing USP7 in the nucleus. However, due to incompatible anti-TRIM32 and anti-USP7 antibodies for in vivo stainings, we were unable to validate this hypothesis experimentally.

In summary, these findings suggest that TRIM32, USP7 and c-Myc exist in a complex and that TRIM32 and USP7 may antagonize each other in the nucleus of proliferating type C cells of the SVZ.

### TRIM32 and USP7 partially co-localize in NSCs and neurons

Since the expression pattern of USP7 in the neural lineage has not been studied so far, we further investigated its subcellular localization in neural progenitor cells and neurons in vitro and in vivo and compared it to that of TRIM32.

**Fig. 4:**
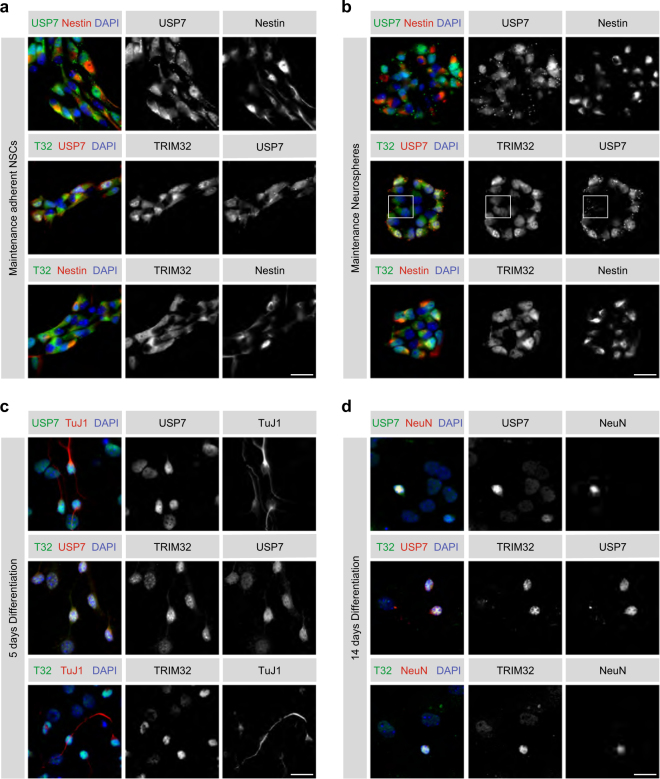
TRIM32 and USP7 co-localize in NSCs and neurons in vitro. **a** Immunostainings of adherent NSCs cultivated under maintenance conditions with the indicated markers. Scale bar = 20 µm. **b** Immunostainings of neurospheres cultivated under maintenance conditions with the indicated markers. Scale bar = 20 µm. Boxed area shows cells with nuclear TRIM32 but cytoplasmic USP7. **c**, **d** Immunostainings of NSCs 5 days (**c**) or 14 days (**d**) after the induction of neuronal differentiation with the indicated markers. Scale bars = 20 µm; *N* ≥ 3

For analyzing USP7 expression in the neural lineage in vitro, adherent primary NSCs isolated from E12.5–E14.5 mouse embryos were stained for USP7 and TRIM32. Consistent with our previous results, the majority of NSCs displayed cytoplasmic TRIM32 [13], while a small fraction of cells, typically located at the borders of the NSC clusters, additionally exhibited nuclear TRIM32 (Fig. 4a). Interestingly, the same expression pattern was observed for USP7 and co-staining of both proteins revealed that cells with nuclear TRIM32 also exhibited nuclear USP7, while those expressing TRIM32 exclusively in the cytoplasm likewise displayed exclusive USP7 expression in the cytoplasm (Fig. 4a). These in vitro observations indicate, that TRIM32 and USP7 may also co-localize in vivo, strengthening our hypothesis of TRIM32 and USP7 being expressed in the same subset of proliferative type C cells in the adult mouse SVZ.

USP7 and TRIM32 displayed a similar expression pattern in neurosphere cultures, which were also derived from E12.5–E14.5 mouse embryos [19]. Also here, cells in the centre of the sphere -corresponding to rather immature cells- usually showed exclusive cytoplasmic TRIM32 and USP7 expression, while cells at the border of the sphere -corresponding to more mature cells- additionally expressed TRIM32 and USP7 in the nucleus (Fig. 4b). Interestingly, in some of the spheres, we observed cells with weak nuclear TRIM32 expression that lacked nuclear USP7 expression, suggesting that the nuclear translocation of TRIM32 may precede that of USP7 in vitro (Fig. 4b). When subjecting adherent NSCs to neuronal differentiation for 5 or 14 days, virtually all of the cells displayed USP7 and TRIM32 in the nucleus (Fig. 4c and d). Of note, cells co-stained with the neuronal markers TuJ1 and NeuN exhibited a considerably stronger nuclear signal for TRIM32 or USP7, compared to cells being negative for TuJ1 and NeuN (Fig. 4c, d). These findings suggest, that both TRIM32 and USP7 become upregulated during the process of neuronal differentiation. However, since c-Myc is already absent at that stage, we assume that the nuclear co-expression of TRIM32 and USP7 has an additional function beyond c-Myc regulation in neurons.

**Fig. 5:**
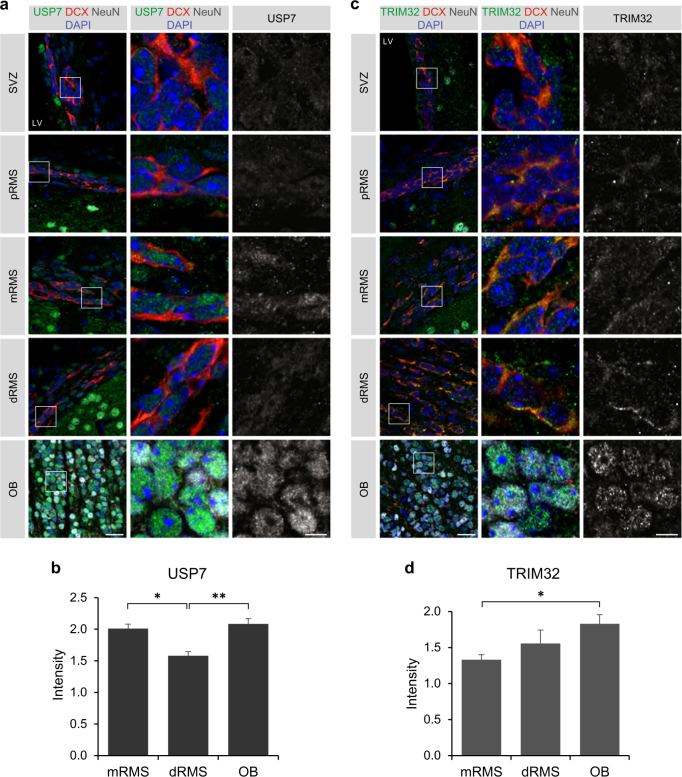
TRIM32 and USP7 show distinct expression patterns in neuroblasts of the distal RMS in vivo. **a**–**c** Immunostainings of adult mouse brain sections with the indicated antibodies. Images were taken from the indicated regions of the SVZ-RMS-OB system. The right panels show a higher magnification of the boxed areas. Scale bars = 20 µm; for high magnifications, 5 µm; *N* = 3. **b**–**d** Quantification of the staining intensity for USP7 (**b**) and TRIM32 (**d**) in the middle RMS, distal RMS and OB, normalized to the background of each section (mean ± SEM; *N* = 3; *t*-test, **p < 0.01; *p < 0.05). pRMS proximal RMS, mRMS middle RMS, dRMS distal RMS

To confirm the neural progenitor culture based observations in vivo, the expression analysis was extended to the adult mouse SVZ-RMS-OB system. Immature neuroblasts in the SVZ and proximal RMS, as identified by DCX co-staining, displayed only weak cytoplasmic and nuclear staining of USP7, while more mature neuroblasts in the middle RMS showed increased expression levels of USP7 (Fig. 5a). Interestingly, a downregulation of USP7 seemed to occur in neuroblasts of the dorsal RMS compared to those in the middle RMS (Fig. 5a, b). In contrast, NeuN + neurons within the OB displayed a strong signal for USP7 in the nucleus (Fig. 5a, b), indicating that USP7 expression becomes upregulated again, when neurons mature.

Consistent with our previous results [18], TRIM32 was only weakly expressed in the cytoplasm of immature neuroblasts of the SVZ and proximal RMS, similar to USP7 (Fig. 5c). However, we observed a continuous upregulation of TRIM32 during the progression of neuroblasts from the middle RMS through the distal RMS to the OB, along with the higher degree of maturity of those neuroblasts (Fig. 5c, d). TRIM32 reached its highest expression levels in NeuN + neurons of the OB, which was accompanied by nuclear translocation of TRIM32 (Fig. 5c, d), in line with our previous findings [18]. These data demonstrate that TRIM32 and USP7 show overlapping expression patterns during early and late stages of neuronal differentiation, while they show distinct expression patterns during the intermediate phase (dorsal RMS). It is tempting to speculate that this dynamic expression pattern of TRIM32 and USP7 mediates c-Myc stabilization in the early phase, its degradation in the intermediate phase and has an additional, c-Myc-unrelated function at later stages of neuronal differentiation.

Furthermore, while TRIM32 expression was barely detectable in astrocytes (Supplementary Fig. 2b) [18], we observed a strong expression for USP7 in astrocytic fibres within the SVZ and proximal RMS (Supplementary Fig. 2a), indicating that USP7 and TRIM32 may play additional independent roles in astrocytes.

### USP7 stabilizes c-Myc by antagonizing its TRIM32-mediated polyubiquitination

**Fig. 6:**
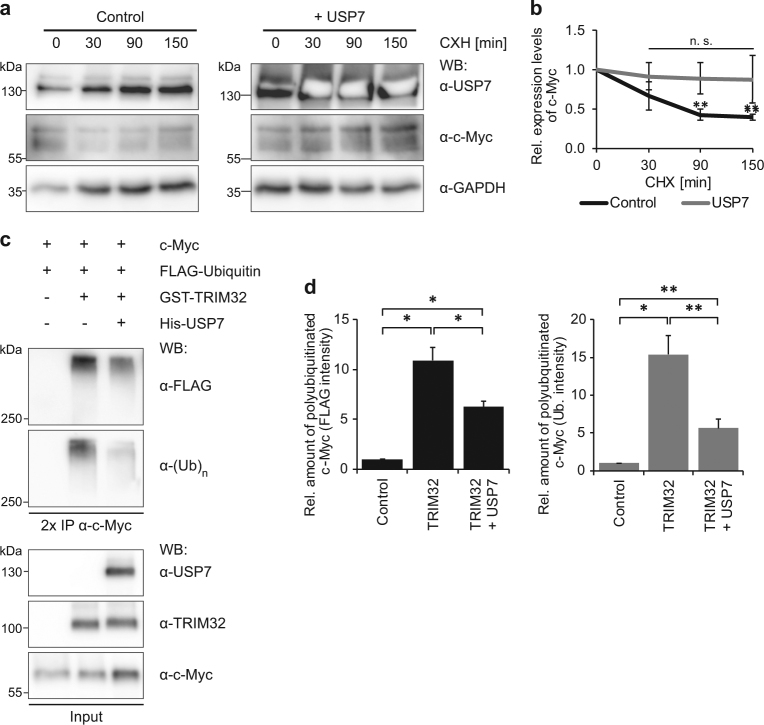
USP7 stabilizes c-Myc levels by antagonizing TRIM32 ubiquitination activity. **a** Cycloheximide treatment of HEK293T cells transfected for 48 h with plasmids expressing FLAG-USP7 or an empty vector as control. Endogenous c-Myc levels were analyzed at 0, 30, 90 and 150 min after cycloheximide treatment by western blot with an anti-c-Myc antibody. GAPDH western blots were used as loading control. **b** Quantification of endogenous c-Myc levels in the presence or absence of overexpressed USP7 as shown in (**a**), normalized to their respective time point 0 (mean ± SEM; *N* = 5 independent experiments; Mann–Whitney *U* test or *t* test, **p < 0.01). **c** c-Myc in vitro deubiquitination assay using recombinant proteins. E1 and E2 (UbcH5a) enzymes were incubated with the indicated components followed by 2 × IP of c-Myc with an anti-c-Myc antibody. Ubiquitination of c-Myc was detected by immunoblotting with an anti-FLAG antibody, directed against the FLAG-tag of ubiquitin, as well as with an antibody directed against mono-and-polyubiquitinylated conjugates (anti-(Ub)_*n*_). **d** Quantification of polyubiquitinated c-Myc levels in FLAG and (Ub)_*n*_ blots shown in (**c**), normalized to the control (mean ± SEM; *N* ≥ 3 independent experiments; Mann–Whitney *U* test or *t* test, **p < 0.01; *p < 0.05). WB western blot, CHX cycloheximide, IP immunoprecipitation

In order to determine the dynamics of the stabilizing effect of USP7 towards c-Myc, which we observed before (Fig. 3a), HEK293T cells were treated with cycloheximide to block new protein synthesis. In HEK293T cells transfected with an empty vector as control, c-Myc was rapidly degraded upon cycloheximide treatment (Fig. 6a, b). In contrast, overexpression of USP7 led to a stabilization of c-Myc levels (Fig. 6a, b), presumably through deubiquitination of c-Myc.

To provide evidence that USP7 is indeed able to deubiquitinate c-Myc and thereby to antagonize TRIM32, an in vitro deubiquitination assay was performed using recombinant c-Myc, GST-TRIM32 and His-USP7 proteins. Since TRIM32 autoubiquitinates itself [13], c-Myc was precipitated twice to ensure its exclusive isolation and to exclude the possibility of contamination with autoubiquitinated TRIM32. Staining the c-Myc 2 × IP blots against the FLAG-tag of Ubiquitin as well as with an antibody-detecting mono- and polyubiquitinated conjugates (α-(Ub)_n_) revealed a prominent high-molecular weight smear in the presence of TRIM32 (Fig. 6d, e), confirming our previous results that TRIM32 robustly polyubiquitinates c-Myc [13, 14, 20, 21]. Interestingly, this ubiquitination smear was significantly reduced in the presence of USP7 (Fig. 6d, e), demonstrating that USP7 is indeed able to reverse the TRIM32-mediated polyubiquitination of c-Myc by deubiquitination.

We were able to validate these observations in vivo by precipitating c-Myc twice from HEK293T cells overexpressing c-Myc, HA-Ubiquitin, GFP-TRIM32 and FLAG-USP7 or the catalytically inactive mutant FLAG-USP7-CS (Supplementary Fig. 3a and b). Similar to the in vitro deubiquitination assay (Fig. 6d, e), overexpression of TRIM32 led to a strong increase in the amount of polyubiquitinated c-Myc (Supplementary Fig. 3a and b). This polyubiquitination was strongly reduced upon co-expression of USP7, but not by co-expression of USP7-CS (Supplementary Fig. 3a and b), further strengthening our findings of USP7 being a deubiquitinase for c-Myc.

Finally, we analyzed whether USP7 also antagonizes the autoubiquitination activity of TRIM32 by performing a TRIM32 in vitro deubiquitination assay. 2 × IP of TRIM32 after incubation with His-USP7 did not show any reduction in the polyubiquitination smear resulting from TRIM32 autoubiquitination (Supplementary Fig. 3c and d), indicating that USP7 does not deubiquitinate TRIM32.

### USP7 activity is required for NSC maintenance

**Fig. 7:**
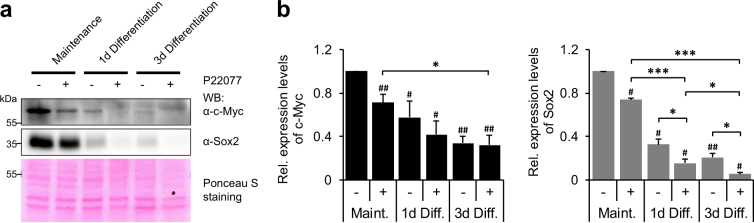
Blocking USP7 activity leads to reduced c-Myc and Sox2 levels in differentiating NSCs. **a** Neurospheres under maintenance conditions as well as 1 day and 3 days after the induction of neuronal differentiation were treated with the USP7 inhibitor P22077 or DMSO as control. Endogenous c-Myc and Sox2 levels were analyzed by western blot with specific antibodies. Ponceau S staining was used as loading control. **b** Quantification of endogenous c-Myc and Sox2 levels in (**a**), normalized to DMSO-treated neurospheres under maintenance conditions (mean ± SEM; *N* ≥ 4 independent experiments; Mann–Whitney *U* test or *t* test, ***p < 0.005; **p < 0.01; *p < 0.05). WB western blot

In order to examine, whether the above described regulation of c-Myc by USP7 has any influence on NSC fate, we blocked USP7 activity in neurospheres under maintenance and neuronal differentiation conditions by using the USP7 inhibitor P22077. Neuronal differentiation of neurospheres treated with DMSO as control resulted in a strong downregulation of c-Myc as well as of the NSC marker Sox2 (Fig. 7), in line with previous reports [22, 23]. USP7 inhibitor treatment enhanced the observed downregulation of c-Myc even further (Fig. 7), demonstrating that USP7 activity is required to stabilize c-Myc also in NSCs. Most importantly, inhibition of USP7 also led to a significant decrease in Sox2 levels in neurospheres both under maintenance and differentiation conditions (Fig. 7), suggesting that the USP7 function is necessary for maintaining NSC fate and preventing premature neuronal differentiation.

## Discussion

In the present study we demonstrate, that the E3 ligase TRIM32 and the DUB USP7 antagonize each other in the post-translational regulation of c-Myc, thereby providing a potential mechanism for cell fate determination in NSCs. These findings are in concordance with the hypothesis proposed by Huang and colleagues, that each SCTF is controlled by at least a pair of E3 ligase and DUB and that the net balance of ubiquitination and deubiquitination determines cell fate [6, 7].

The E3 ligase TRIM32 has been shown to possess ubiquitinating activity for numerous physiological targets, thereby regulating a variety of biological processes. For instance, in the neural lineage, TRIM32 ubiquitinates the Myc family members N-Myc [24] and c-Myc [13, 14, 20, 21], thereby targeting both proteins for proteasomal degradation and inducing differentiation. Accordingly, TRIM32 becomes upregulated upon neuronal differentiation and translocates to the nucleus [18, 19] (Supplementary Fig. 1a). There, it can ubiquitinate the SCTF c-Myc, leading to its degradation (Supplementary Fig. 1b and c) [13, 14]. Since we observed a subpopulation of proliferative type C cells in the SVZ, that already weakly expressed TRIM32 in the nucleus (Fig. 1), we sought for DUBs that may antagonize TRIM32 activity towards c-Myc, and thereby prevent the induction of premature neuronal differentiation in these cells.

Consistent with TRIM32 functioning as an E3 ligase, we identified various ubiquitination-associated proteins as potential TRIM32 interaction partners (Fig. 2a) in a previously performed mass spectrometry screen [19], such as proteasomal proteins and several E2 enzymes such as UBE2N, which has been shown to interact with TRIM32 earlier [25] (Fig. 2b). Also, several DUBs were identified among these ubiquitination-associated proteins (Fig. 2b), of which we decided to focus on the DUB USP7, due to its earlier implication in the regulation of neural progenitor cell maintenance [6, 7]. By controlling their ubiquitination levels, USP7 regulates the stability, function or localization of various proteins involved in diverse cellular processes such as DNA repair, transcription, epigenetic regulation, immune response, cell cycle and mitosis. The role of USP7 has been best characterized with respect to tumor biology, due to its upregulation in various cancer cells and its regulation of the tumor suppressor genes p53, PTEN and FOXO [7, 26]. Furthermore, USP7 seems to be indispensable for brain development, as specific deletion of USP7 in neural cells causes apoptosis and severe brain malformations in mouse embryos, ultimately leading to neonatal lethality [27].

Besides its expression in neuroblasts, USP7 expression was further observed in a subset of proliferating type C cells of the SVZ in vivo (Fig. 3b), similar to TRIM32 (Fig. 1). We therefore hypothesize, that in these cells USP7 antagonizes TRIM32 in targeting c-Myc for ubiquitin-dependent proteasomal degradation, thereby preventing neuronal differentiation. This hypothesis is further corroborated by the fact that USP7 indeed is able to deubiquitinate and stabilize c-Myc polyubiquitinated by TRIM32 (Figs. 6 and 7).

Various studies have demonstrated, that the SCTF c-Myc is able to inhibit the terminal differentiation of multiple cell types, by, for example, repressing the expression of differentiation-inducing genes [22]. Interestingly, by binding to its promoter, c-Myc inhibits the transcription of the microRNA *Let-7a* [28, 29], which is a potent inducer of neuronal differentiation in NSCs [14]. Thus, by stabilizing c-Myc, USP7 prevents the expression of neuronal differentiation-inducing genes, such as *Let-7a*, and maintains NSC fate. Consistently, inhibition of USP7 in NSCs undergoing neuronal differentiation led not only to a downregulation of c-Myc, but also of the NSC marker Sox2 (Fig. 7), a step that is required to allow for the initiation of neuronal differentiation [30, 31].

Our findings are in agreement with an earlier study proposing a similar mechanism by which USP7 regulates neural progenitor cell maintenance [6, 7]. According to that study, USP7 stabilizes the SCTF REST, which is a transcriptional repressor of neuronal differentiation. During neuronal differentiation, REST becomes ubiquitinated by the E3 ligase complex SCF_β-TrCP_ and subsequently degraded by the proteasome, thereby promoting neuronal differentiation due to released repression of differentiation-associated genes [32, 33]. Through deubiquitination, USP7 antagonizes the SCF_β-TrCP_-mediated ubiquitination of REST, thereby preventing its proteasomal degradation and promoting neural progenitor cell maintenance [6, 7]. Thus, USP7 is able to ensure the maintenance of NSCs also by regulating SCTFs other than c-Myc and by antagonizing E3 ligases other than TRIM32.

Another recent example of a SCTF, which is regulated by USP7, is the Myc family member N-Myc [34]. USP7 was shown to deubiquitinate N-Myc, thereby stabilizing it in human neuroblastoma cells. Consistently, overexpression of USP7 in human neuroblastoma cells led to upregulation of N-Myc [34], which is in agreement to our findings of upregulated c-Myc in HEK293T cells after overexpression of USP7 (Figs. 3a and 6a, b). However, in contrast to our findings, Tavana et al. did not observe any effect of USP7 on c-Myc ubiquitination or protein levels in human neuroblastoma cells [34]. These discrepancies might be due to cell-type-specific differences between HEK293T cells and human neuroblastoma cells or due to the different spatial and temporal expression patterns, which have been described for N-Myc and c-Myc [22, 35]. Interestingly, also TRIM32 has recently been shown to regulate N-Myc in human neuroblastoma cells by ubiquitinating it at spindle poles during mitosis, thereby targeting it for proteasomal degradation and inducing asymmetric cell division [24]. These data suggest, that the antagonistic role of TRIM32 and USP7 may not be restricted to the regulation of c-Myc, but may also be relevant for other Myc family members, such as N-Myc, or even for additional SCTFs.

## Materials and methods

### Material and plasmids

For immunolabeling and protein biochemical methods, the following antibodies have been used: anti-TRIM32-3150 (Gramsch Laboratories), anti-TRIM32-3149 (Gramsch Laboratories), anti-TRIM32-M09 (Abnova), anti-TRIM32-GS (Genescript), anti-TRIM32-1137 (Gramsch Laboratories) [13, 18–20], anti-USP7 (Novus Biologicals), anti-c-Myc N-262 (Santa Cruz), anti-mono-and-polyubiquitinylated conjugates (FK2) (Enzo Life Sciences), anti-FLAG M2 (Sigma), anti-enhanced green fluorescent protein (anti-EGFP) (Abcam), anti-α-Tubulin (Sigma-Aldrich), anti-Ki67 (BD Biosciences), anti-Mash1 (BD Biosciences), anti-Nestin (BD Biosciences), anti-Tuj1 (BioLegend), anti-Doublecortin X (Millipore), anti-Neuronal Nuclei (anti-NeuN) (Millipore) and anti-GFAP (Millipore). As secondary antibodies Alexa-fluorophore-conjugated antibodies (Invitrogen) were used for immunofluorescence stainings and horseradish peroxidase-coupled antibodies (GE Healthcare) for immunoblotting. DNA was counterstained using Hoechst 33258 (Invitrogen).

The following plasmids were used: pEGFP-N1 (Clontech Laboratories), pcDNA3-GFP-TRIM32 (kindly provided by Dr. Germana Meroni), pEGFP-TRIM32-ΔRING [14], pcDNA3-c-Myc (kindly provided by Dr. Martin Eilers), pBSK-HA-Ubiquitin (kindly provided by Dirk Bohrmann), pQFlag-USP7-WT-puroR and pQFlag-USP7-CS-puroR (kindly provided by Dr. Goedele Maertens).

### Mice

C57BL/6 N mice were obtained from Charles River. Breeding, maintenance and experimental procedures of all mice were performed in accordance with institutional and national guidelines and regulations.

### Cell culture

Primary NSCs were isolated from C57BL/6 N mouse brains at embryonic day (E) 12.5-14.5 for adherent as well as for neurosphere cultures. For adherent cultures, primary NSCs were cultivated and passaged as described previously [36, 37]. Briefly, adherent NSCs were maintained on poly-D-Lysine (Sigma)-coated 10-cm polystyrene tissue culture dishes in DMEM/Ham’s F12 medium (PAA) supplemented with 10 ng/mL EGF (Peprotech), 10 ng/mL bFGF-2 (Peprotech), 1 × N2 (Invitrogen), L-Glutamine (PAA), and Penicillin/Streptomycin (PAA). Neuronal differentiation was induced by exchanging 50% of the maintenance medium by DMEM/Ham’s F12 medium (PAA) supplemented with 10 ng/mL bFGF-2 (Peprotech), 1 × N2 (Invitrogen), 1 × B27 (Invitrogen), L-Glutamine (PAA), and Penicillin/Streptomycin (PAA).

For neurosphere cultures, primary NSCs were cultivated and passaged as described previously [19]. Briefly, neurospheres were maintained on uncoated 10-cm polystyrene tissue culture dishes in the same maintenance medium as adherent NSCs. For immunocytochemical staining, neurospheres were seeded onto Poly-ornithine (Sigma)-/Laminin (Sigma)-coated coverslips.

HEK293T cells were cultivated on uncoated 10-cm polystyrene tissue culture dishes in DMEM (Sigma) supplemented with 10% heat-inactivated FCS (PAA), L-Glutamine (PAA) and Penicillin/Streptomycin (PAA). For transfection experiments, HEK293T cells were seeded onto poly-D-Lysine (Sigma)-coated 10-cm polystyrene tissue culture dishes and transfected using Turbofect (Fermentas) according to manufacturer’s instructions. Each transfection experiment was conducted at least three independent times.

### Immunoprecipitation and western blot

For immunoprecipitation assays and Western blot analyses, HEK293T cells were lysed 48 h after transfection with nuclear lysis buffer (50 mM TRIS (pH 7.5), 0.5 M NaCl, 1% NP-40, 1% DOC, 0.1% SDS, 2 mM EDTA, and Complete protease inhibitor (Roche) in ddH_2_O) for 30 min at 4 °C, followed by centrifugation for 30 min at 13,000 r.p.m. and 4 °C. Protein concentrations were determined using a BCA protein assay kit (Thermo Scientific) according to manufacturer’s instructions. Samples were adjusted to equal protein concentrations and the percentage of SDS was reduced to 0.07% by adding TRIS/EDTA (10 mM TRIS (pH 7.5), 1 mM EDTA). A fraction of the cell lysate was mixed with sample buffer and boiled at 95 °C for 5 min, while the remaining lysate was incubated with the precipitation antibody for 4 h at 4 °C followed by incubation with protein-G agarose beads (GE Healthcare) overnight at 4 °C. Bound proteins were eluted by boiling the samples in protein sample buffer for 15 min at 95 °C and collected by centrifugation at 13,000 r.p.m. for 5 min. The supernatants were subjected to SDS-PAGE and western blotting as described previously [19]. In brief, IP samples and lysates were size-separated by electrophoresis on 7.5% Novex SDS-PAGE gels according to manufacturer’s instructions (Invitrogen). Equal amounts of blotted protein were verified by staining the membranes with Ponceau S (Sigma). Analysis of Western Blots was performed using Adobe Photoshop and ImageJ software.

### c-Myc and TRIM32 in vitro deubiquitination assay

Recombinant c-Myc was obtained from Abcam, recombinant GST-TRIM32 protein was obtained from Abnova and recombinant His-USP7 was obtained from BostonBiochem. Prior to the c-Myc deubiquitination assay, an ubiquitination reaction was performed as described previously [13, 21]. Briefly, c-Myc and GST-TRIM32 were incubated with activated E1 enzyme, E2 enzyme and FLAG-Ubiquitin (BostonBiochem) at room temperature (RT) for 1 h. The ubiquitination reaction was stopped by the addition of 2.5 mM EDTA. This was followed by the deubiquitination reaction, for which His-USP7 was added to one of the samples for 1 h at RT. The samples were incubated with a specific anti-c-Myc antibody and protein-G agarose beads as described above. Bound proteins were eluted by boiling the samples in 1% SDS for 15 min and collected by centrifugation at 13000 rpm for 5 min. The supernatants were subjected to a second round of immunoprecipitation as described above, followed by SDS-PAGE and western blotting. In order to detect ubiquitinated proteins, western blots were incubated using an anti-FLAG as well as an anti-mono-and-polyubiquitinylated conjugates antibody.

The TRIM32 deubiquitination assay was performed as described for the c-Myc deubiquitination assay (see above). First, an ubiquitination reaction was performed, during which recombinant GST-TRIM32 was incubated with activated E1 enzyme, E2 enzyme and FLAG-Ubiquitin (BostonBiochem) at room temperature (RT) for 1 h. The ubiquitination reaction was stopped by the addition of 2.5 mM EDTA. This was followed by the deubiquitination reaction, for which His-USP7 was added to one of the samples for 1 h at RT. A 2 × immunoprecipitation was performed as described above using a specific anti-TRIM32 antibody. In order to detect ubiquitinated proteins, western blots were incubated using an anti-FLAG as well as an anti-mono-and-polyubiquitinylated conjugates antibody.

### c-Myc in vivo Deubiquitination assay

HEK293T cells were lysed in nuclear lysis buffer 48 h after transfection as described above. Samples were adjusted to equal protein concentrations and the percentage of SDS was reduced to 0.07% by adding TRIS/EDTA. A fraction of the cell lysate was mixed with sample buffer and boiled at 95 °C for 5 min, while the remaining lysate was incubated with the precipitation antibody for 4 h at 4 °C followed by incubation with protein-G agarose beads (GE Healthcare) overnight at 4 °C. Bound proteins were eluted by boiling the samples in 1% SDS at 95 °C for 15 min. After centrifugation at 13,000 r.p.m. for 5 min the supernatants were subjected to a second round of immunoprecipitation as described above. The next day, samples were boiled in protein sample buffer at 95 °C for 15 min and centrifuged at 13,000 r.p.m. for 5 min. The supernatants were subjected to SDS-PAGE and Western blotting as described above.

### Subcellular fractionation

For subcellular fractionation neurospheres under maintenance conditions as well as 3 day-old neurons were lysed in 300 µl subcellular fractionation buffer (250 mM Sucrose, 20 mM HEPES (pH 7.4), 10 mM KCl, 1.5 mM MgCl_2_, 1 mM EDTA, 1 mM EGTA, 1 mM dithiothreitol (DTT) and Complete protease inhibitor (Roche) in ddH_2_O). Lysates were passed 10 times through a 20 G needle followed by a 25 G needle. The samples were incubated on ice for 20 min, after which they were centrifuged at 3000 r.p.m. for 5 min to obtain the nuclear pellet. The supernatant was centrifuged again for 5 min at 8000 r.p.m. and the supernatant of that step represented the cytosolic fraction. The nuclear pellet was washed by adding 2 ml of subcellular fractionation buffer, dispersed with a pipette and passed 10 times through a 25 G needle. After centrifugation at 3000 r.p.m. for 10 min, the nuclear pellet was resuspended in 150 µl of subcellular fractionation buffer containing 10% glycerol and 0.1% SDS and sonicated briefly. The cytosolic and nuclear fractions were adjusted to equal protein concentrations and boiled in protein sample buffer for 5 min at 95 °C. The samples were subjected to SDS-PAGE and Western blotting as described above. Antibodies against Histone H1 and GAPDH were used as markers for the nuclear and cytosolic fraction, respectively.

### CHX treatment

HEK293T cells were treated with 50 µg/ml cycloheximide (CHX, Sigma) 48 h after transfection and harvested after 0, 30, 90 and 120 min. The cells were lysed in nuclear lysis buffer and analyzed by Western blotting as described above.

### USP7 inhibitor treatment

Neurospheres cultivated under maintenance conditions were treated with 8 µM of the USP7 Inhibitor P22077 (Calbiochem) or with DMSO as control. After 48 h of treatment, one fraction of the neurospheres was lysed in nuclear lysis buffer. The remaining cells were plated onto Poly-ornithin-/Laminin-coated plates and differentiated into neurons. Neurons were lysed in nuclear lysis buffer after 1 day and 3 days of neuronal differentiation. All samples were adjusted to equal protein concentrations and boiled in protein sample buffer for 5 min at 95 °C. The samples were subjected to SDS-PAGE and Western blotting as described above.

### Immunocytochemistry

For immunocytochemical stainings, cells were fixed using 4% paraformaldehyde in 120 mM PBS, pH 7.4 (4% PFA/PBS) for 15 min followed by permeabilisation for 3 min at 4 °C using 0.05% Triton X-100 in PBS. Cells were blocked in 10% FCS in PBS for 1 h at RT and incubated overnight with the primary antibodies diluted in blocking solution. On the following day, the cells were washed and incubated with the secondary antibodies diluted in blocking solution. Images were collected with a Zeiss epifluorescence microscope and image analysis was performed with ZEN lite software (Zeiss), Adobe Photoshop and ImageJ software.

### Perfusion, sectioning and immunohistochemistry of free-floating sections

Mice were deeply anesthetized and intracardially perfused with 50 ml PBS followed by 50 ml 4% PFA/PBS solution. Brains were dissected and post-fixed overnight in 4% PFA/PBS solution at 4 °C. Sagittal brain sections of 40 µm were prepared by using a vibratome (Leica VT 1000 S) and blocked for at least 1 h in TBS (0.1 M Tris, 150 mM NaCl, pH 7.4) containing 0.5% Triton X-100, 0.1% Na-Azide, 0.1% Na-Citrate and 5% normal goat serum. Immunostainings were performed by incubating the sections with the primary antibody diluted in blocking solution for 48 h at 4 °C on a shaker, followed by incubation with the secondary antibody diluted in blocking solution for 2 h at RT. Images were collected with a Zeiss confocal microscope and image analysis was performed with ZEN lite software (Zeiss), Adobe Photoshop and ImageJ software.

### Mass spectrometry analysis

Mass spectrometry was performed as previously described [19]. Briefly, TRIM32 was precipitated from NSCs cultivated under either maintenance or neuronal differentiation conditions. Proteins, that co-precipitated with TRIM32, were analyzed by Nanoflow LC-MS/MS as described previously [19, 38]. The statistical analysis for the identification of proteins that were significantly enriched in the differentiating NSC samples was carried out as described in [19]. These proteins were considered as potential interaction candidates. The Gene Ontology (GO) enrichment analysis was performed as previously described [19] but with an adjusted *P* value cutoff below 0.01. Protein–protein interaction data were retrieved from MetaCore (GeneGo Inc. [39]) using proteins that were enriched in the NSC samples in comparison to the negative control, and were present in ubiquitin-related categories among enriched GO categories. The network was visualized in Cytoscape [40]. Proteins common among enriched ubiquitin-related GO categories were visualized with the igraph R package [41].

## Electronic supplementary material

Supplementary Information

Supplementary Figure 1

Supplementary Figure 2

Supplementary Figure 3

## References

[1] Gage FH (2000). Mammalian neural stem cells. Science.

[2] Komander D, Rape M (2012). The ubiquitin code. Annu Rev Biochem.

[3] Hershko A, Ciechanover A (1998). The ubiquitin system. Annu Rev Biochem.

[4] Pickart CM, Eddins MJ (2004). Ubiquitin: structures, functions, mechanisms. Biochim Biophys Acta.

[5] Komander D, Clague MJ, Urbe S (2009). Breaking the chains: structure and function of the deubiquitinases. Nat Rev Cell Biol.

[6] Huang Z, Wu Q, Guryanova OA, Cheng L, Shou W, Rich JN (2011). Deubiquitylase HAUSP stabilizes REST and promotes maintenance of neural progenitor cells. Nat Cell Biol.

[7] Huang Z, Zhou W, Bao S (2011). Role of deubiquitylase HAUSP in stem cell maintenance. cell cycle (Georgetown. Tex).

[8] Ming GL, Song H (2011). Adult neurogenesis in the mammalian brain: significant answers and significant questions. Neuron.

[9] Zhao C, Deng W, Gage FH (2008). Mechanisms and functional implications of adult neurogenesis. Cell.

[10] Adhikary S, Eilers M (2005). Transcriptional regulation and transformation by Myc proteins. Nat Rev Mol Cell Biol.

[11] Meyer N, Penn LZ (2008). Reflecting on 25 years with MYC. Nat Rev Cancer.

[12] Davis AC, Wims M, Spotts GD, Hann SR, Bradley A (1993). A null c-myc mutation causes lethality before 10.5 days of gestation in homozygotes and reduced fertility in heterozygous female mice. Genes Dev.

[13] Hillje AL, Worlitzer MM, Palm T, Schwamborn JC (2011). Neural stem cells maintain their stemness through protein kinase C zeta-mediated inhibition of TRIM32. Stem Cells.

[14] Schwamborn JC, Berezikov E, Knoblich JA (2009). The TRIM-NHL protein TRIM32 activates microRNAs and prevents self-renewal in mouse neural progenitors. Cell.

[15] Ke Y, Zhang EE, Hagihara K, Wu D, Pang Y, Klein R (2007). Deletion of Shp2 in the brain leads to defective proliferation and differentiation in neural stem cells and early postnatal lethality. Mol Cell Biol.

[16] Fults D, Pedone C, Dai C, Holland EC (2002). MYC expression promotes the proliferation of neural progenitor cells in culture and in vivo. Neoplasia.

[17] Gonzalez-Cano L, Hillje AL, Fuertes-Alvarez S, Marques MM, Blanch A, Ian RW (2013). Regulatory feedback loop between TP73 and TRIM32. Cell death & Dis.

[18] Hillje AL, Pavlou MA, Beckmann E, Worlitzer MM, Bahnassawy L, Lewejohann L (2013). TRIM32-dependent transcription in adult neural progenitor cells regulates neuronal differentiation. Cell death & Dis.

[19] Nicklas S, Okawa S, Hillje AL, Gonzalez-Cano L, Del Sol A, Schwamborn JC (2015). The RNA helicase DDX6 regulates cell-fate specification in neural stem cells via miRNAs. Nucleic Acids Res.

[20] Nicklas S, Otto A, Wu X, Miller P, Stelzer S, Wen Y (2012). TRIM32 regulates skeletal muscle stem cell differentiation and is necessary for normal adult muscle regeneration. PLoS ONE.

[21] Bahnassawy L, Perumal TM, Gonzalez-Cano L, Hillje AL, Taher L, Makalowski W (2015). TRIM32 modulates pluripotency entry and exit by directly regulating Oct4 stability. Sci Rep.

[22] Eilers M, Eisenman RN (2008). Myc’s broad reach. Genes Dev.

[23] Zhang S, Cui W (2014). Sox2, a key factor in the regulation of pluripotency and neural differentiation. World J Stem Cells.

[24] Izumi H, Kaneko Y (2014). Trim32 facilitates degradation of MYCN on spindle poles and induces asymmetric cell division in human neuroblastoma cells. Cancer Res.

[25] Napolitano LM, Jaffray EG, Hay RT, Meroni G (2011). Functional interactions between ubiquitin E2 enzymes and TRIM proteins. Biochem J.

[26] Nicholson B, Suresh Kumar KG (2011). The multifaceted roles of USP7: new therapeutic opportunities. Cell Biochem Biophys.

[27] Kon N, Zhong J, Kobayashi Y, Li M, Szabolcs M, Ludwig T (2011). Roles of HAUSP-mediated p53 regulation in central nervous system development. Cell Death Differ.

[28] Chang TC, Yu D, Lee YS, Wentzel EA, Arking DE, West KM (2008). Widespread microRNA repression by Myc contributes to tumorigenesis. Nat Genet.

[29] Sampson VB, Rong NH, Han J, Yang Q, Aris V, Soteropoulos P (2007). MicroRNA let-7a down-regulates MYC and reverts MYC-induced growth in Burkitt lymphoma cells. Cancer Res.

[30] Bylund M, Andersson E, Novitch BG, Muhr J (2003). Vertebrate neurogenesis is counteracted by Sox1-3 activity. Nat Neurosci.

[31] Graham V, Khudyakov J, Ellis P, Pevny L (2003). SOX2 functions to maintain neural progenitor identity. Neuron.

[32] Guardavaccaro D, Frescas D, Dorrello NV, Peschiaroli A, Multani AS, Cardozo T (2008). Control of chromosome stability by the beta-TrCP-REST-Mad2 axis. Nature.

[33] Westbrook TF, Hu G, Ang XL, Mulligan P, Pavlova NN, Liang A (2008). SCFbeta-TRCP controls oncogenic transformation and neural differentiation through REST degradation. Nature.

[34] Tavana O, Li D, Dai C, Lopez G, Banerjee D, Kon N (2016). HAUSP deubiquitinates and stabilizes N-Myc in neuroblastoma. Nat Med.

[35] Dang C. V. (2013). MYC, Metabolism, Cell Growth, and Tumorigenesis. Cold Spring Harbor Perspectives in Medicine.

[36] Conti L, Cattaneo E (2010). Neural stem cell systems: physiological players or in vitro entities?. Nat Rev Neurosci.

[37] Conti L, Pollard SM, Gorba T, Reitano E, Toselli M, Biella G (2005). Niche-independent symmetrical self-renewal of a mammalian tissue stem cell. PLoS Biol.

[38] van den Berg DL, Snoek T, Mullin NP, Yates A, Bezstarosti K, Demmers J (2010). An Oct4-centered protein interaction network in embryonic stem cells. Cell Stem Cell.

[39] Nikolsky Y, Ekins S, Nikolskaya T, Bugrim A (2005). A novel method for generation of signature networks as biomarkers from complex high throughput data. Toxicol Lett.

[40] Shannon P, Markiel A, Ozier O, Baliga NS, Wang JT, Ramage D (2003). Cytoscape: a software environment for integrated models of biomolecular interaction networks. Genome Res.

[41] Csardi G, Nepusz T (2006). The igraph software package for complex network research. Int J Complex Syst.

