# Supplementary material for: A complex of the ubiquitin ligase TRIM32 and the deubiquitinase USP7 balances the level of c-Myc ubiquitination and thereby determines neural stem cell fate specification
Source: Cell Death Differ. 2018 Jun 13;26(4):728–40. doi: 10.1038/s41418-018-0144-1 (PMC6460386; doi:10.1038/s41418-018-0144-1)
Supplement: Supplementary file 1 — Supplementary Information [file 41418_2018_144_MOESM1_ESM.docx]

**A complex of the ubiquitin ligase TRIM32 and the deubiquitinase USP7 balances the level of c-Myc ubiquitination and thereby determines neural stem cell fate specification**

**Running title: TRIM32 and USP7 determine neural stem cell fate**

**- Supplementary information -**

Sarah Nicklas^1,2^, Anna-Lena Hillje^1,2^, Satoshi Okawa^3^, Ina-Maria Rudolph^2^, Franziska Melanie Collmann^2^, Thea van Wüllen^1,2^, Antonio del Sol^3^, Jens C. Schwamborn^1,2^*

**Supplementary Figure Legend**

**Supplementary Figure 1: TRIM32 translocates to the nucleus upon neuronal differentiation and destabilizes c-Myc.**

(**a**) Subcellular fractionation of neurospheres under maintenance conditions and 3 days after the induction of neuronal differentiation. Cytosolic and nuclear TRIM32 was detected with a specific anti-TRIM32 antibody. Antibodies against GAPDH and Histone H1 were used as markers for the cytosolic and nuclear fraction, respectively. N = 4. Abbreviations: cyt, cytosolic; nuc, nuclear; WB, Western blot. (**b**) Western blot analysis of c-Myc expression levels in HEK293T cells transfected for 72 h with plasmids expressing c-Myc, HA-Ubiquitin and GFP-TRIM32 as indicated. An α-GAPDH Western blot was used as loading control. N = 4. Abbreviations: WB, Western blot. (**c**) Quantification of c-Myc levels in the presence or absence of overexpressed GFP-TRIM32 as shown in (**b**), normalized to the control (mean ± SEM; N = 4 independent experiments; Mann-Whitney U test, *p < 0.05).

**Supplementary Figure 2: USP7 is expressed in astrocytic fibres *in vivo*.**

(**a**) + (**b**) Immunostainings of adult mouse brain sections with the indicated antibodies. Images were taken from the SVZ and proximal RMS region. Scale bars = 20 µm; N = 3. Abbreviations: pRMS, proximal RMS.

**Supplementary Figure 3: USP7 antagonizes TRIM32 ubiquitination activity towards c-Myc *in vivo*, but not the autoubiquitination of TRIM32 *in vitro*.**

(**a**) c-Myc *in vivo* deubiquitination assay in HEK293T cells transfected with the indicated constructs. c-Myc was immunoprecipitated twice (2x IP) with an anti-c-Myc antibody. Ubiquitination of c-Myc was detected by immunoblotting with an anti-HA antibody, directed against the HA-tag of ubiquitin, as well as with an antibody directed against mono- and-polyubiquitinylated conjugates (anti-(Ub)_n_). N ≥ 5. (**b**) Quantification of polyubiquitinated c-Myc levels in HA and (Ub)_n_ blots shown in (**a**), normalized to the control (mean ± SEM; N ≥ 5 independent experiments; Mann-Whitney U test or t-test, ***p < 0.005). (**c**) TRIM32 *in vitro* deubiquitination assay using recombinant proteins. E1 and E2 (UbcH5a) enzymes were incubated with the indicated components followed by 2x IP of TRIM32 with an anti-TRIM32 antibody. Ubiquitination of TRIM32 was detected by immunoblotting with an anti-FLAG antibody, directed against the FLAG-tag of ubiquitin, as well as with an antibody directed against mono-and-polyubiquitinylated conjugates (anti-(Ub)_n_). N ≥ 3. Abbreviations: IP, immunoprecipitation; WB, Western Blot. (**d**) Quantification of polyubiquitinated c-Myc levels in FLAG and (Ub)_n_ blots shown in (**c**), normalized to the control (mean ± SEM; N ≥ 3 independent experiments).
