# Supplementary figures and images for: A complex of the ubiquitin ligase TRIM32 and the deubiquitinase USP7 balances the level of c-Myc ubiquitination and thereby determines neural stem cell fate specification
Source: Cell Death Differ. 2018 Jun 13;26(4):728–40. doi: 10.1038/s41418-018-0144-1 (PMC6460386; doi:10.1038/s41418-018-0144-1)

**a**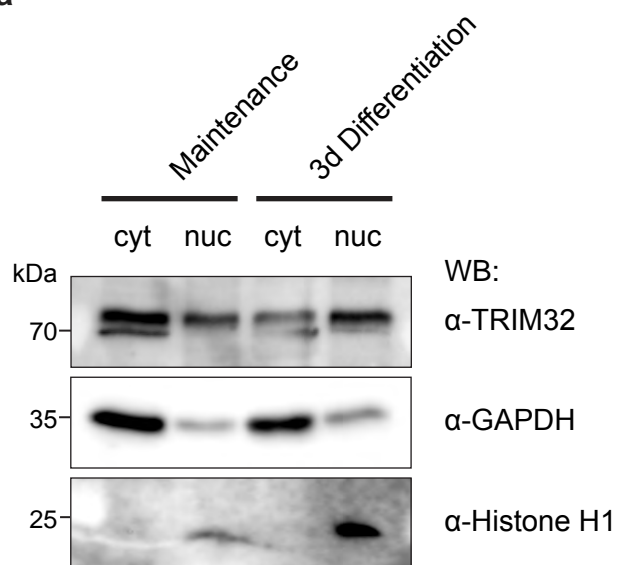**b**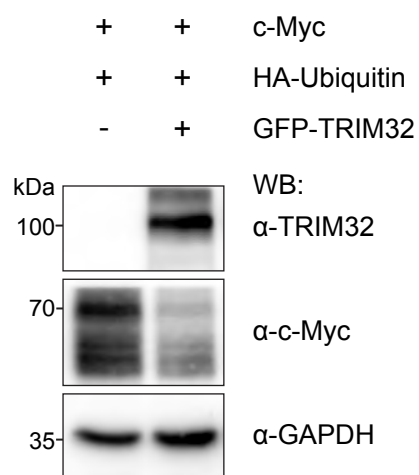**c**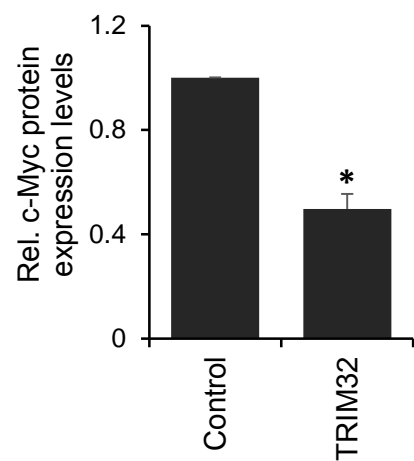

Supplement: Supplementary file 2 — Supplementary Figure 1 [file 41418_2018_144_MOESM2_ESM.pdf]

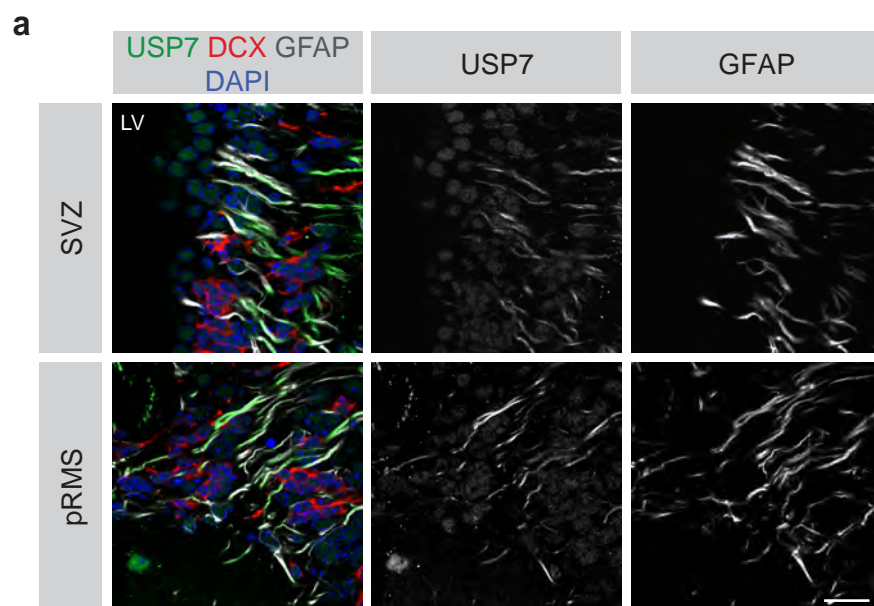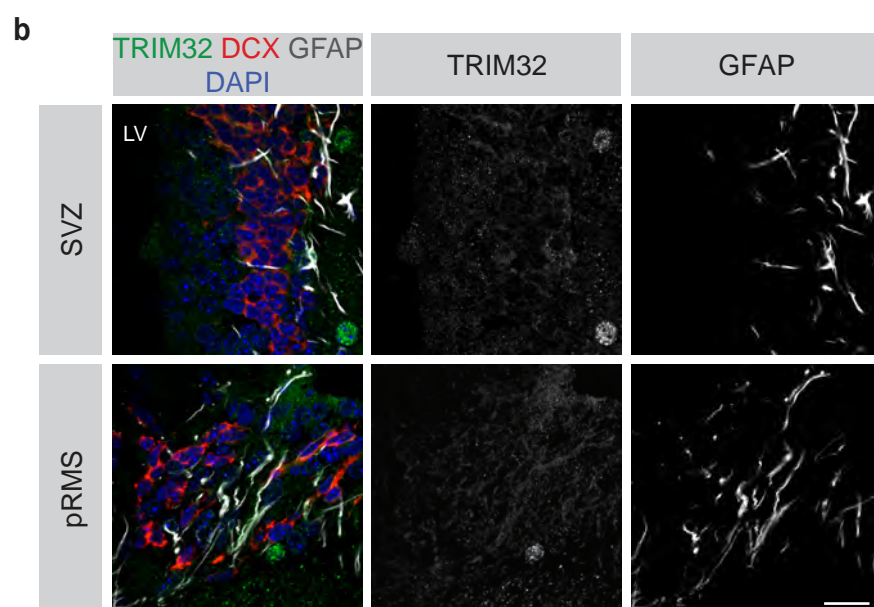

Supplementary Figure 2

Supplement: Supplementary file 3 — Supplementary Figure 2 [file 41418_2018_144_MOESM3_ESM.pdf]

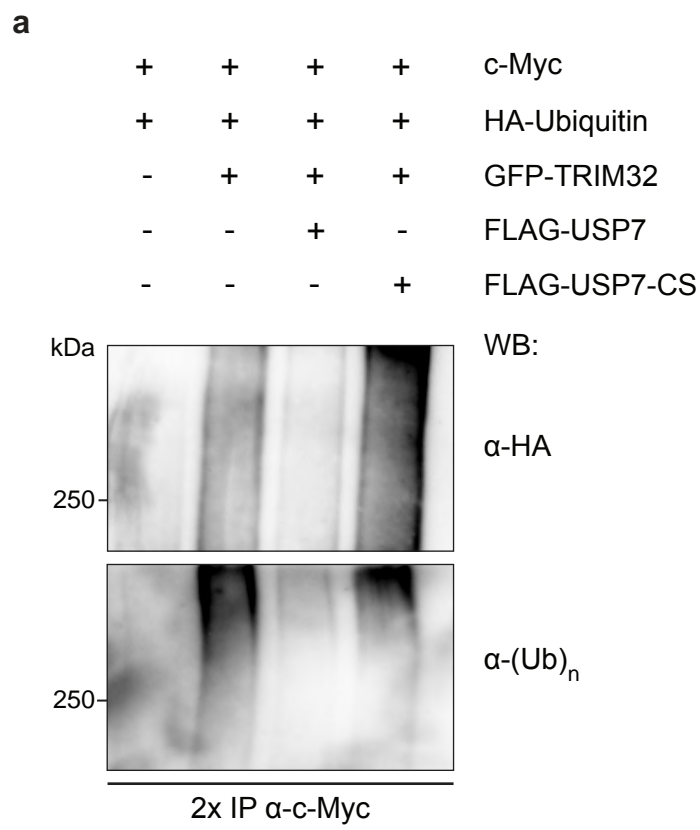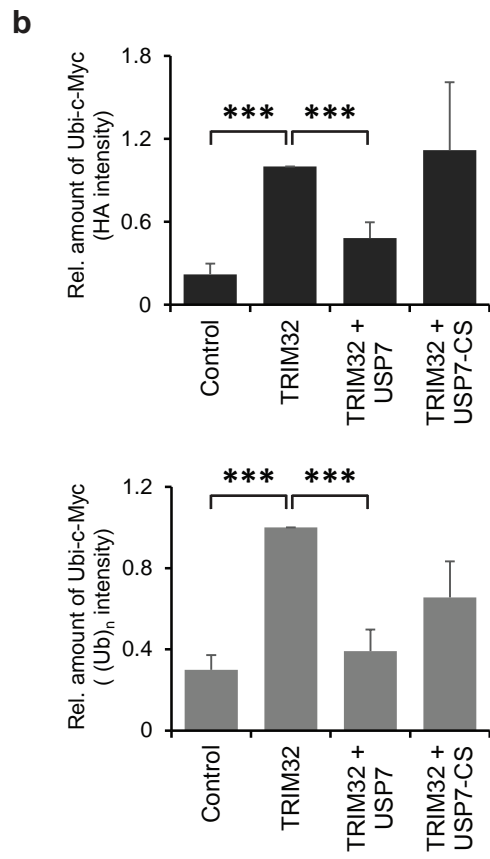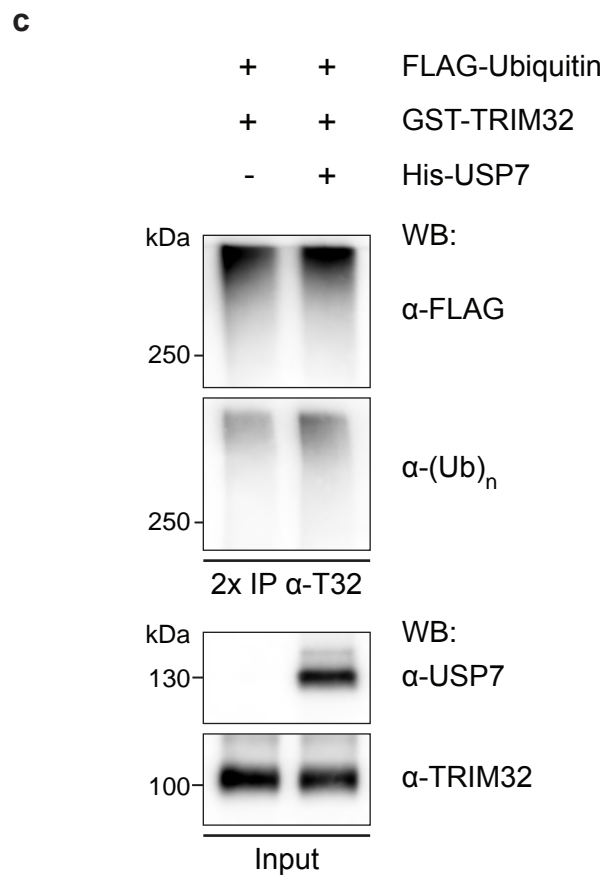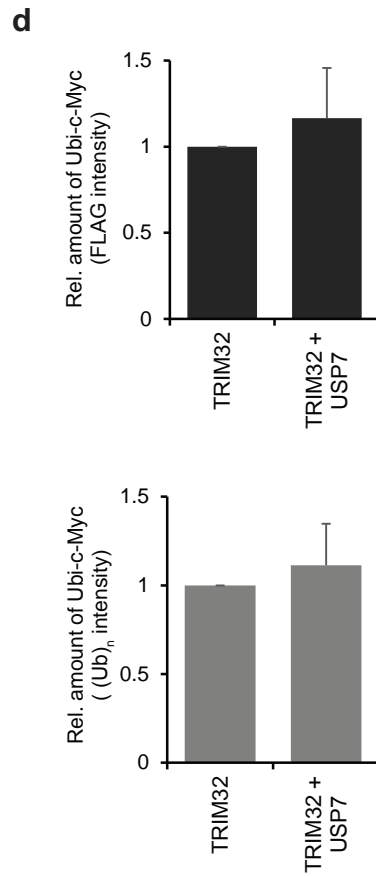

Supplement: Supplementary file 4 — Supplementary Figure 3 [file 41418_2018_144_MOESM4_ESM.pdf]
